# Malicious traffic prediction model for ResNet based on Maple-IDS dataset

**DOI:** 10.1371/journal.pone.0322000

**Published:** 2025-05-13

**Authors:** Qingfeng Li, Boyu Wang, Xueyan Wen, Yuao Chen

**Affiliations:** 1 Network Information Center, Northeast Forestry University, Heilongjiang, China; 2 College of Computer and Control Engineering, Northeast Forestry University, Heilongjiang, China; National University of Sciences and Technology NUST, PAKISTAN

## Abstract

In light of the increasing threat posed by cyberattacks, it is imperative for organizations to accurately identify malicious network traffic. However, the imbalance among various attack categories diminishes the accuracy of model predictions. To address this issue, we propose the Maple-IDS dataset as an innovative solution. We utilize DPDK along with its zero-copy (ZC) technology and BPF compiler to compile filtering rules. Additionally, a headless client is employed to generate control traffic, thereby preventing overfitting. Our data collections are sourced from a variety of operating systems and middleware platforms, ensuring broad applicability and relevance. By comparing our dataset with the CIC-IDS-2017 dataset, we achieve a more balanced representation of attack data, which enhances the model’s learning performance. To tackle the challenges of low accuracy and slow convergence speed in existing network security situation predictions, we propose a network situation awareness prediction model that integrates a residual network with an improved attention mechanism. This model leverages the attention mechanism to assign greater weight to abnormal data, thereby facilitating the accurate identification of anomalies within large data streams. Furthermore, the residual network accelerates convergence speed, enhances the model’s expressive capability, and improves the efficiency of rapid response to attacks. Experimental results indicate that the accuracy of predicting attack data flows reaches an impressive 99.83%, which significantly aids in the early detection of network security threats and enables preemptive measures to maintain normal network operations.

## 1 Introduction

The emergence of cybersecurity situational awareness is closely linked to the rapid advancements in information technology and the growing issue of network security. As the Internet becomes more widespread and technologies like big data and cloud computing continue to progress, network security has become a pressing concern. Incidents such as network attacks and data breaches have become increasingly common, posing significant risks and losses to individuals, businesses, and governments. In most cases, defense only detects the existence of the attack, but knows nothing about when the attack occurred, the victim point, the attacke’s true identity, intention, etc. In addition, complex data makes information processing more difficult. These problems all It will become a challenge to trace the origin and strengthen situational awareness and defense capabilities.Cybersecurity situational awareness involves the real-time monitoring and analysis of network events, such as attacks and unusual accesses, that could jeopardize network security. By utilizing monitoring and analysis technologies, this approach aims to detect security threats early on and implement timely countermeasures to protect the network’s security and stability. It involves the collection, analysis, and processing of various types of network information to provide real-time insights into network security status and to identify and respond to potential threats and attacks promptly.

Situational awareness technology was initially proposed by Endsley in 1988, who defined it as the identification and understanding of environmental factors under specific spatial and temporal conditions, along with the prediction of future trends [1]. Endsley structured the situational awareness model into three levels: situational extraction, situational comprehension, and situational prediction. In 1999, Tim Bass introduced a theory concerning cybersecurity situational awareness [2]. Originally rooted in the military domain for assessing military environment and readiness, the concept of situational awareness has since been adopted in various sectors such as healthcare, transportation, and stock prediction networks, eventually extending into the realm of cybersecurity. Predictive modeling in cybersecurity involves gathering metrics from diverse sources. Cybersecurity situational awareness entails forecasting the future state of a network based on past posture assessments [3]. Given that cybersecurity posture is a time series data, exhibiting certain correlations between preceding and subsequent data points, the notion of cybersecurity situational awareness and prediction is indeed viable.

In recent years, many industries have embraced deep learning as a key direction for development, and the field of cybersecurity situational awareness is no exception. Deep learning, in comparison to traditional methods, has shown effectiveness in approximating and fitting nonlinear time series data. To enhance network posture prediction in cybersecurity assessments, researchers have combined various models to leverage the strengths of each. Given the time-sensitive nature of computer network security, with new vulnerabilities emerging continuously as computers operate, rapid perception of network posture is crucial. This study presents a cybersecurity situational awareness model that integrates the attention mechanism and residual network structure. The CIC-IDS-2017 dataset serves as the baseline data, while the Maple-IDS dataset [4] is generated in the experimental environment. The model’s validity is confirmed through comparative tests.

In this study, VM images with vulnerabilities were obtained from the Vulnhub website, and traffic was captured using tcpdump. Docker Compose was utilized to define and execute multi-container Docker applications, including components like a network traffic generator, marking program, and BPF filter. Subsequently, filtering rules were developed for the BPF filter and loaded into the kernel to be applied to network traffic. To handle mixed black and white traffic, a marking program was created to differentiate and label various types of traffic, which was then integrated into the Docker Compose file.

The research findings of this study are as follows:

1) The use of attention mechanism to improve data filtering and focus on important information.
2) The implementation of residual network algorithm to prevent model overfitting, enable deeper layers, and speed up convergence.
3) Dataset pre-processing to minimize subjective bias in model data processing outcomes.

This study introduces a network security posture prediction model that combines ResNet with an enhanced attention mechanism. The attention mechanism is optimized to enhance the model’s performance, and experimental analysis quantitatively assesses prediction accuracy and the optimal number of applicable layers. Results show that compared to traditional models, this approach improves accuracy quickly, accelerates model convergence, prevents overfitting, and effectively categorizes attack labels. This enables proactive network repair and maintenance to minimize network losses.

Choosing ResNet over traditional CNNs or LSTMs enables the training of deeper networks through residual connections, resulting in improved performance. The strength of LSTMs lies in their capacity to manage long-term dependencies in sequences, a feature that may not be fully leveraged in data that lacks such characteristics. Conversely, CNNs, due to their relatively fixed structure, are less adaptable than ResNet for modifications tailored to various environments. Consequently, this paper chooses to utilize ResNet for network data analysis.

The paper is structured into four main sections. The second section covers the techniques related to the model, while the third section outlines the components of the model. Finally, the fourth section provides a summary of the experimental results.

## 2 Related work

Cybersecurity situational awareness [5] is the process of assessing the network posture based on current environmental factors to predict the future state of the network. Predicting the network security posture is crucial for identifying potential security risks early and evaluating the impact of these threats, enabling network security managers to understand the current network status.

In the realm of traditional models, literature [6] utilized the Markov Multi-stage Transferable Belief Model (MM-TBM) to effectively address the multi-stage nature of cyber attacks and enhance cyber defense through multi-stage data fusion. Literature [7]proposed the utilization of Support Vector Machines (SVMs) which not only significantly improved prediction accuracy post-training but also reduced training time.Moreover, in the context of deep learning-based approaches, literature [8] introduced a posture prediction model that combines a temporal convolutional network with Transformer to effectively tackle long-term prediction modeling of time series data.Literature [9] presented a network security situational awareness method called MSCB-BPNN, which integrates the variable scale chaotic bat (MSCB) algorithm with BP neural network (BPNN). This model demonstrates clear advantages over existing methods such as BPNN, SA-BPNN, and GA-BPNN. Although the aforementioned models have achieved notable accomplishments in the field of cybersecurity situational awareness, they still possess certain limitations. The Markov model relies on the Markov property assumption, which may not align with specific practical scenarios. Support Vector Machines (SVM), being inherently a binary classification model, exhibit low computational efficiency with large sample sizes. The Transformer, on the other hand, excessively depends on large-scale data, while the Backpropagation Neural Network (BPNN) is prone to issues such as gradient explosion or vanishing.Due to differences in network data format and large data volumes, coupled with interspersed attack traffic [10], implementing an attention mechanism [11,12] can effectively increase the weight of attack data and exhibit strong performance in handling network attacks.

Before the emergence of residual networks, deep networks faced performance degradation as the number of layers increased. This degradation hindered effective weight adjustment in previous layers, leading to higher training errors and poor results in training and testing, known as degradation. ResNet introduced ‘short-circuit’ connections, transforming the original output *H(x)=F(x)* into *H(x)=F(x)+x*. This allowed for continuous model updates without deteriorating previous training results. However, the performance of a single model structure in cyber situational awareness remains subpar, prompting researchers to explore fusion modeling as a solution for cybersecurity situational prediction.

This paper proposes a network posture prediction model that combines residual structures and an attention mechanism. The attention mechanism, which has been utilized in machine translation, speech recognition, text summarization, and other tasks [13–15], is a versatile and efficient tool for improving the handling of sequential data.

The purpose of this study is to predict potential network attacks by analyzing a large amount of data in a short period of time. Understanding the types of attacks can enhance network stability and mitigate risks. The model utilizes residual structure [16] to accelerate convergence, stabilize performance, and prevent overfitting. Additionally, the attention mechanism prioritizes attack labels in input sequences by assigning increased weights, ultimately enhancing the efficiency and accuracy of the model.

## 3 Design of a network posture prediction model for attention mechanisms based on residual networks

Residual networks have demonstrated significant success in various deep learning tasks, including image classification [17], target detection [18], speech recognition [19], and other domains. It is evident from previous research that residual networks primarily excel in classification tasks. This section introduces the concepts of residual networks and attention mechanisms, followed by the presentation of the model design.

### 3.1 Overview of residual networks

**Fig 1:**
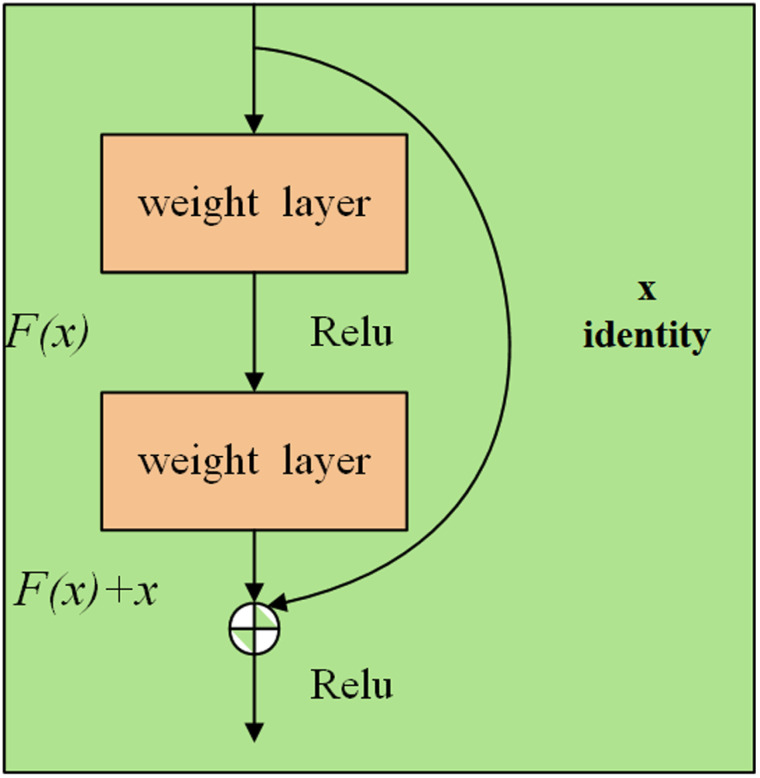
Residual block model.

Simply increasing the depth of the network can result in gradient explosion and vanishing problems in conventional CNNs. These issues are typically addressed through weight regularization or gradient thresholding, but these approaches may cause network degradation. Therefore, ResNet was developed as a solution. Residual networks were initially proposed by Kaiming He [20]et al. in 2015, with the core concept being to address the training challenges of deep networks by introducing residual connections. In traditional neural networks, each layer’s output is derived solely from the previous layer’s output, whereas in residual networks, each layer’s output is a combination of the previous layer’s output and the input to that layer. Residual networks allow part of the input to directly skip one or more layers and pass to deeper layers by adding shortcut connections, thereby promoting direct backpropagation of gradients without adding additional parameters or computational complexity. Fig 1 illustrates an example of a residual block model.

The residual block formula for a single layer is shown below:


$$\begin{array}{c} y=F\left(\text{x},w_{i}\right)+x \end{array}$$ (1)

where *F(x,W*_*i*_*)* is the output of the layer with weight Wi, x is the input and y is the output. This residual block converts several layers within the deep network from learning a constant mapping function to learning a residual function.By reducing the model to a shallow network, the conversion formula is:


$$\begin{array}{c} H\left(x\right)=F\left(x\right)+x\to F\left(x\right)=H\left(x\right)-x \end{array}$$ (2)

When the residual network updates the parameters of a node, the equation *H(x) = F(x) + x* ensures that the result after chain derivation, avoids issues like gradient vanishing or explosion. This is because the presence of 1 on the left side alters the chain derivation law from multiplication to addition, maintaining a continuous parameter update process. Consequently, even with small changes in the derivation parameter on the right side, the node parameters do not suffer from gradient-related problems [21]. This results in a constant mapping H(x) = x when F(x) = 0, enabling the neural network to efficiently fit the data.

### 3.2 Overview of attention mechanisms

**Fig 2:**
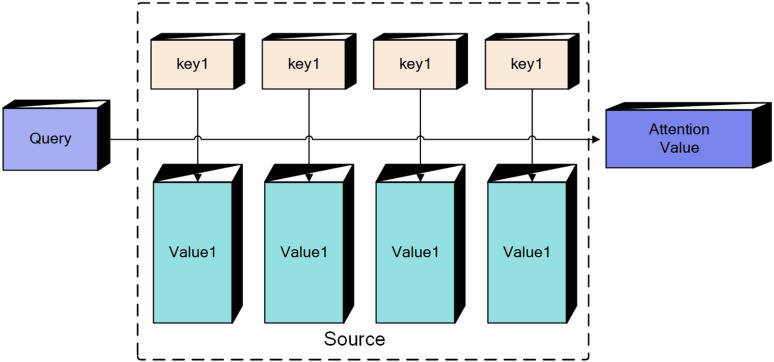
chematic representation of attention mechanisms.

The Attention Mechanism is a scientific principle that mimics the human attention mechanism and is primarily used to enhance performance in processing sequential data [22]. The core concept of the attention mechanism involves assigning weights to input data during processing to improve the model’s performance and generalization. As illustrated in Fig 2, the constituent elements of the data set can be conceptualized as a series of pairs. In this context, when a specific element, referred to as the Query, is identified in the Target, the similarity or correlation between the Query and each Key is calculated. This process yields a weight coefficient for the corresponding Value associated with each Key. Subsequently, the weighted sum of these Values is computed, resulting in the final Attention value.

The introduction of the channel attention mechanism enhances the focus on specific features, allowing the model to better understand the local intricacies within the data. This mechanism involves passing input features through both a global average pooling (AvgPool) layer and a global maximum pooling (MaxPool) layer. Global Average Pooling retains overall information of the features, while Global Maximum Pooling extracts highly representative features.

The process is shown in the following equation:


$$\begin{array}{c} M_{c}=\sigma \left(MLP\left(AvgPool\left(f\right)\right)\right)+\;MLP\left(MaxPool\left(F\right)\right) \end{array}$$ (3)

A spatial attention mechanism is proposed to reduce unnecessary feature expression. Each feature point extracted from the feature channel undergoes global averaging and maximum pooling. The results are then stacked and fused with a 7×7 convolutional kernel to generate weighted information, as shown in the following equation:


$$\begin{array}{c} M_{s}\left(F^{\prime }\right)=\sigma \left(f^{7\times 7}\left[Avgpool\left(F^{\prime }\right);MaxPool\left(F^{\prime }\right)\right]\right) \end{array}$$ (4)

### 3.3 Residual network based modeling incorporating the attention mechanism

**Fig 3:**
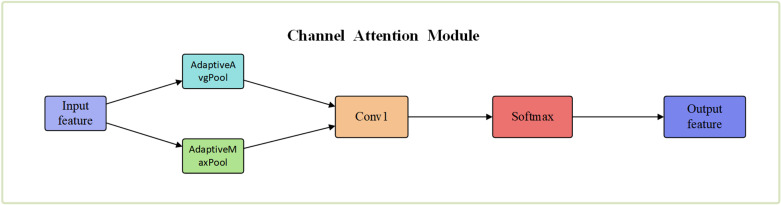
Hannel attention module.

**Fig 4:**
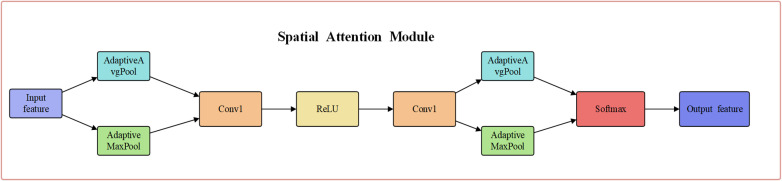
Spatial attention module.

After preprocessing the data, the attention mechanism used to allocate attention to the attack data in the input data is CBAM. CBAM consists of two sub-modules: the channel attention module and the spatial attention module. These sub-modules are designed to weight and adjust the features in the channel dimension and spatial dimension, respectively, in order to enhance the characterization ability of the feature representation. The two sub-modules are illustrated in Figs 3 and 4

The model starts with convolution and normalization of the input data and since the input features are one dimensional vectors its expression is as follows:


$$\begin{array}{c} MH=BatchNorm1d\left(Conv1d\left(x\right)\right) \end{array}$$ (5)

The input data, being a one-dimensional tensor, is processed using ReLU activation function followed by CBAM attention mechanism. CBAM is mathematically represented by the following equation:


$$\begin{array}{c} F^{\prime }=M_{c}\left(F\right)⊗F \end{array}$$ (6)

$$\begin{array}{c} {F^{\prime }}^{\prime }=M_{s}\left(F^{\prime }\right)⊗F^{\prime } \end{array}$$ (7)

**Fig 5:**
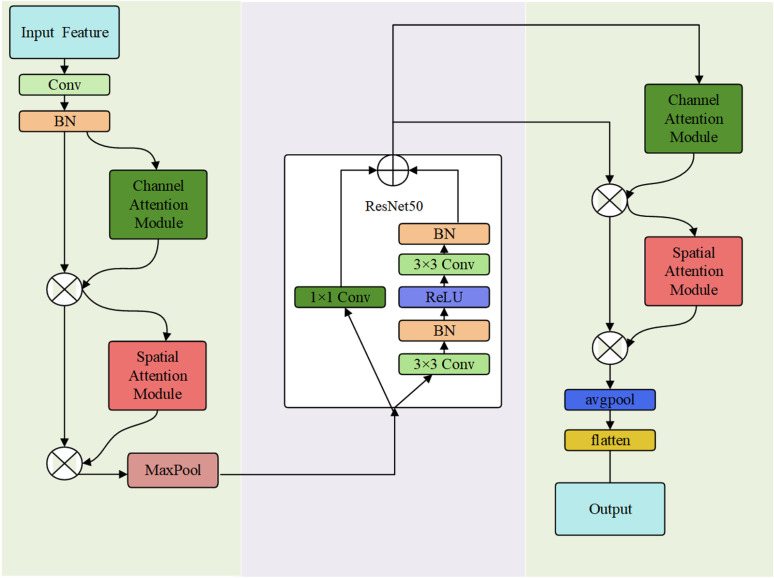
ResNet50-CBAM structure diagram.

The feature tensor F is processed using channel-based attention Mc and spatial-based attention Ms, with element-by-element multiplication denoted by ⊗. The resulting feature maps are denoted as F’ and F’‘, representing the output after channel-based and spatial attention. These output features are then input into the residual structure with multiple layers to enhance the convergence speed of the model. The model structure is illustrated in the following Fig 5.

As illustrated in the table below, we present the pseudocode of the model to provide further detail. This pseudocode outlines the definition of the initialization network layer, the incorporation of the attention mechanism within the network layer, and the specification of the residual module.

**Table:** 

Pseudocode
Class ResNet(nn.Module): def __init__(self, block_type, block_counts, num_classes=1000, include_top=True, groups=1, width_per_group=64): Call the constructor of nn.Module Set instance variables for include_top, groups, and width_per_group Initialize in_channel to 64, which is the output channel count of maxpool and the input channel count for residual blocks Initialize the first convolutional layer (conv1), batch normalization layer (bn1), ReLU activation, channel attention (ca), spatial attention (sa), and max pooling layer (maxpool) For each layer in [1, 2, 3, 4]: Create a layer with the specified block type, channel count, block count, and stride Update in_channel to the output channel count of the created layer If include_top is True: Initialize the adaptive average pooling layer (avgpool) and the fully connected layer (fc) For each module in the network: If the module is a convolutional layer (nn.Conv1d): Initialize its weights using kaiming normal initialization with fan_out mode and relu nonlinearity def _make_layer(self, block_type, channel_count, block_count, stride=1): Initialize downsample to None If stride is not 1 or self.in_channel is not equal to channel_count * block_type.expansion: Create a downsample path using a convolutional layer and batch normalization layer Initialize an empty list to store blocks Append the first block to the list, updating in_channel to the output channel count of the block For the remaining blocks in the layer: Append a block to the list Return a Sequential model containing all the blocks in the layer def forward(self, x): Apply the static layers (conv1, bn1, relu, ca, sa, maxpool) to the input x Apply the dynamic layers (layer1, layer2, layer3, layer4) to x Apply channel attention (ca1) and spatial attention (sa1) to x If include_top is True: Apply the adaptive average pooling layer (avgpool), flatten the output, and apply the fully connected layer (fc) Return the final output

## 4 Results and analysis

### 4.1 Datasets

In the Maple-IDS dataset, traffic configuration is designed based on the behavioral patterns of users, endpoints, and real-world traffic. Maple-IDS dataset configuration is shown in Table 1. By utilizing protocols like HTTP, HTTPS, and SM3/4, we analyze and model users’ access request behavior.In comparison to other datasets, the Maple-IDS dataset includes SSH, RESTful API, gRPC, and WASM protocols, along with various implementations. The dataset was conducted on Windows 10 LTSC and Linux, specifically Debian, Kali, and Rocky Linux distributions. To address the performance bottleneck of the Windows API, libpcap and winpcap were utilized.Utilizing open source technologies on the Linux platform, we successfully employed DPDK with its Zero Copy (ZC) technology, along with the BPF compiler for compiling filtering rules. Subsequently, we utilized CICFlowMeter and a Rust implementation to process pcap/pcapng packets and convert them into usable data.In container and cloud-native scenarios, Sidecar containers are utilized for efficient harvesting of data, preventing duplication of back-origin traffic harvesting within containers. When dealing with CVE and other vulnerability traffic, vulhub and corresponding exploit scripts are employed to generate malicious traffic, while headless clients are used to generate control traffic, thereby preventing overfitting.

**Table 1:** Maple-IDS dataset configuration.

Class	Number
All	2588
BENIGN	1080
DDoS	1386
Openssl_normal	16
Heartbleed	106

The dataset was collected from diverse operating systems and middleware platforms, ensuring broad applicability and relevance. Tools such as libpcap, npcap, DPDK, and ntop were employed to capture traffic data across various environments. These tools are renowned for their efficiency and precision in network traffic capture.

Data was gathered from a variety of network settings, including mirror gateways, sidecar containers, cloud-native environments, datacenters, and Docker bridge networks. This diversity ensures that the dataset reflects a wide spectrum of real-world network scenarios. The dataset includes traffic related to modern Common Vulnerabilities and Exposures (CVEs) and technologies like DNS over HTTPS (DoH) and DNS over QUIC (DoQ). It also covers modern versions of traditional network infrastructure components, making it highly relevant for current security research. Feature engineering was conducted to extract meaningful attributes from the raw data, focusing on those indicative of potential intrusions.

The inclusion of data from various network environments and technologies makes this dataset suitable for analyzing modern network behaviors and threat patterns. Maple-IDS dataset focus on modern CVEs and new network technologies ensures that the dataset is up-to-date with the latest security challenges. By capturing traffic from actual network deployments, the dataset provides realistic scenarios that enhance its applicability in real-world intrusion detection systems. An ongoing process is in place to monitor the dataset and incorporate new data, adapting to emerging threats. This ensures the dataset remains effective for contemporary research.

We utilize PF_RING ZC (Zero Copy) technology to capture original traffic data within the network. PF_RING ZC is a high-performance network data capture solution that efficiently captures network traffic, significantly reducing the number of data copies between the kernel and user space. This reduction minimizes system overhead and enhances the speed and accuracy of data capture. The primary function of PF_RING is to acquire real-time data packet information from the network and store it in pcap/pcapng file format. These files provide detailed records of various data packets, serving as a foundation for subsequent data analysis and processing.

After being persisted to hard disk storage, these files are initially processed using the pcap2para tool. The pcap2rsa tool is specifically designed to extract HTTP parameters and other relevant information from pcap files, facilitating the identification and extraction of communication content within network traffic. This capability is crucial for analyzing scenarios such as cyberattacks. Furthermore, the request data segment of the pcap/pcapng file can be extracted to prepare for subsequent analysis.

The extracted data is processed using HyperScan, a high-performance regular expression matching library that enables rapid pattern matching of extensive text data. In the realm of network security, HyperScan can be employed to identify various attack patterns and features of malicious code within network traffic. By defining a series of regular expression rules pertinent to security threats, HyperScan efficiently screens for potential attacks and abnormal traffic. It extracts key features of network traffic, such as stream duration, number of bytes transmitted, number of packets, and protocol type. These features are then integrated with their corresponding labels (e.g., normal traffic or a specific attack type) to create a structured dataset.

We export the processed and consolidated data to CSV file format using CICFlowMeter, a robust network traffic analysis tool that can export various characteristics and labeling information of network traffic into easily analyzable and processable CSV files. The exported CSV file format is uniform and standardized, facilitating its use across various data analysis and machine learning frameworks. Additionally, users can leverage CICFlowMeter to manually export CSV files with customized columns tailored to their specific research needs, enabling more targeted analysis and accommodating the data requirements of diverse research scenarios.

In comparison to normal traffic, attack traffic constitutes a larger proportion of the dataset, ensuring that it comprehensively reflects the characteristics and behavioral patterns of network attacks. The distribution ratio of various types of attack traffic within the total attack traffic is determined based on their frequency and significance in real-world network attacks, thereby enhancing the dataset’s realism and representativeness.

The distribution of flow durations in the dataset is broad, encompassing both short transient traffic and longer duration sessions. The stream durations of normal traffic are typically associated with specific business operations, such as system updates and file transfers, resulting in a relatively centralized distribution. In contrast, the stream durations of attack traffic exhibit significant variability, depending on the type and intent of the attack. For instance, certain DDoS attacks may persist for extended periods to maintain constant pressure on the target, while rapid scanning attacks tend to have shorter stream durations.

Similar to flow duration, the distribution of packet counts also exhibits diversity. The number of packets in normal flows is influenced by business requirements; for instance, simple web browsing may involve only a small number of packets exchanged, whereas large-scale data transmissions typically consist of a greater number of packets. In the case of attack flows, DDoS attacks are generally characterized by a significant increase in packet counts compared to normal flows. Conversely, some stealthy attacks may involve fewer packets but pose a greater threat.

### 4.2 Experiments based on CIC-IDS-2017

After processing the CIC-IDS-2017 dataset [23,24], the processed data was utilized as the target of the experiment. The dataset consists of a small proportion of abnormal data mixed with a larger amount of normal data, and encompasses 15 types of attacks such as DDoS attack, PortScan, SSH-Patator, among others. The goal was to assess the effectiveness of predicting different types of attacks. Subsequent experiments were carried out based on this dataset.

### 4.3 Evaluation of initial experimental results

**Fig 6:**
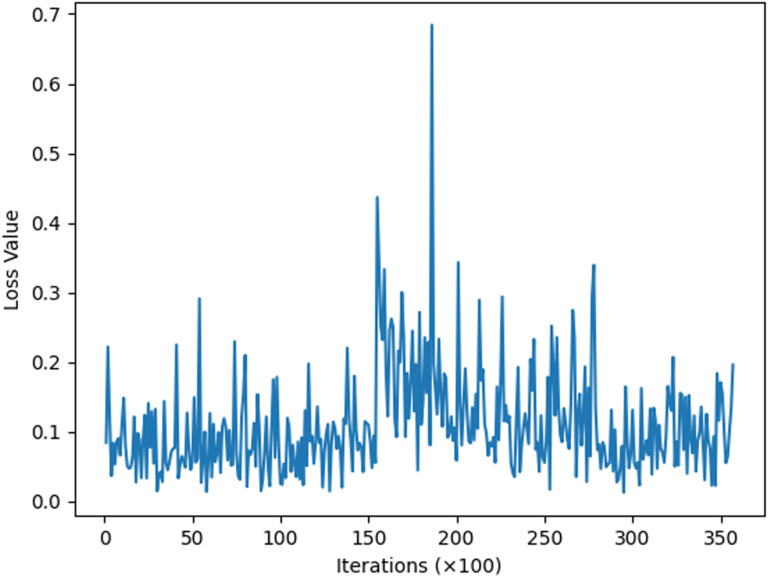
ResNetCBAM34.

**Fig 7:**
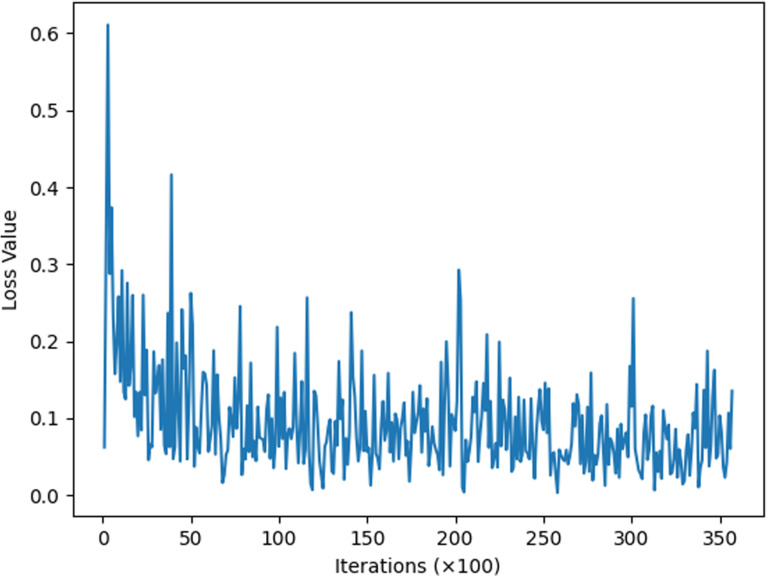
ResNetCBAM50.

**Fig 8:**
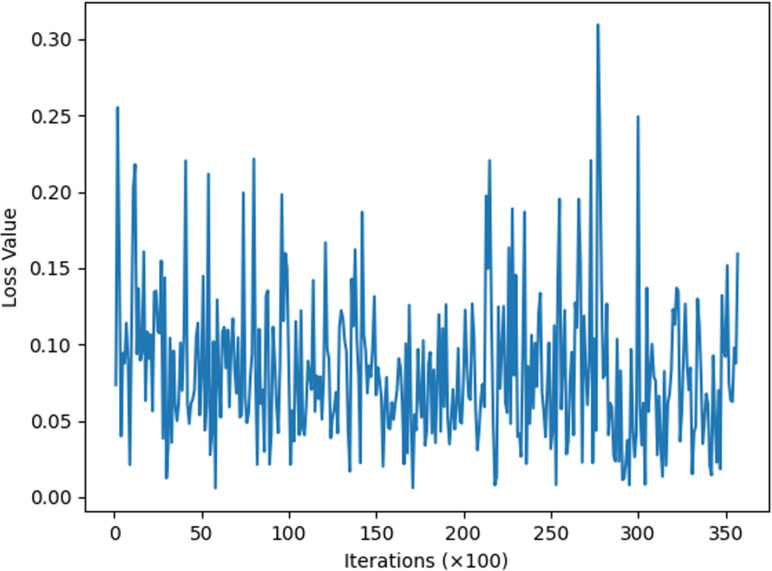
ResNetCBAM101.

This section describes experiments conducted to enhance the model’s situational awareness by incorporating an improved attention mechanism into the traditional residual network. This mechanism allows for the weighting of real attack data during data analysis. The model underwent initial training for two iterations, followed by re-training based on this initial training. Subsequently, loss function images for the 34-layer, 50-layer, and 101-layer models were derived, as depicted in Figs 6–8.

The image above illustrates that, with the same number of iterations, the 34-layer model exhibits fluctuating loss values, while the 50-layer model shows more convergence in its loss function. The 101-layer model demonstrates greater stability, with all loss values falling between 0.0 and 0.3. Although there are differences in loss values across layers, these variances are not very pronounced. The provided data alone does not clearly indicate the optimal number of layers to use, necessitating more detailed data and charts for comparison.

**Fig 9:**
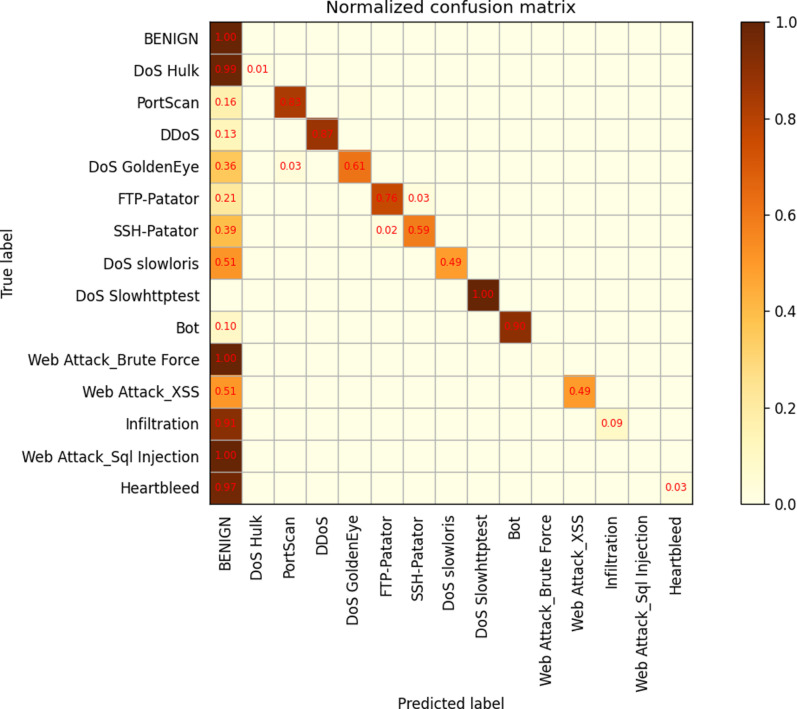
ResNetCBAM34.

**Fig 10:**
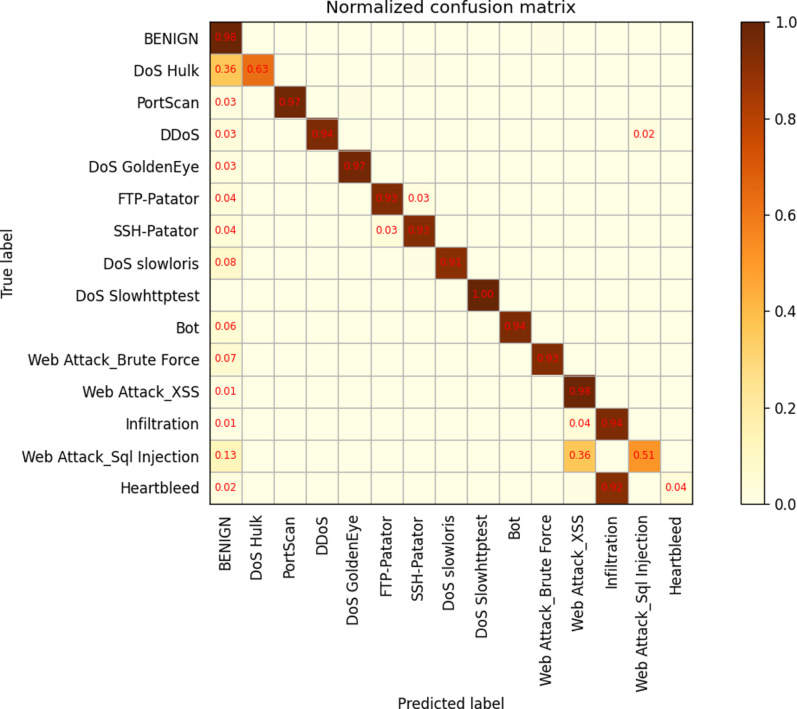
ResNetCBAM50.

**Fig 11:**
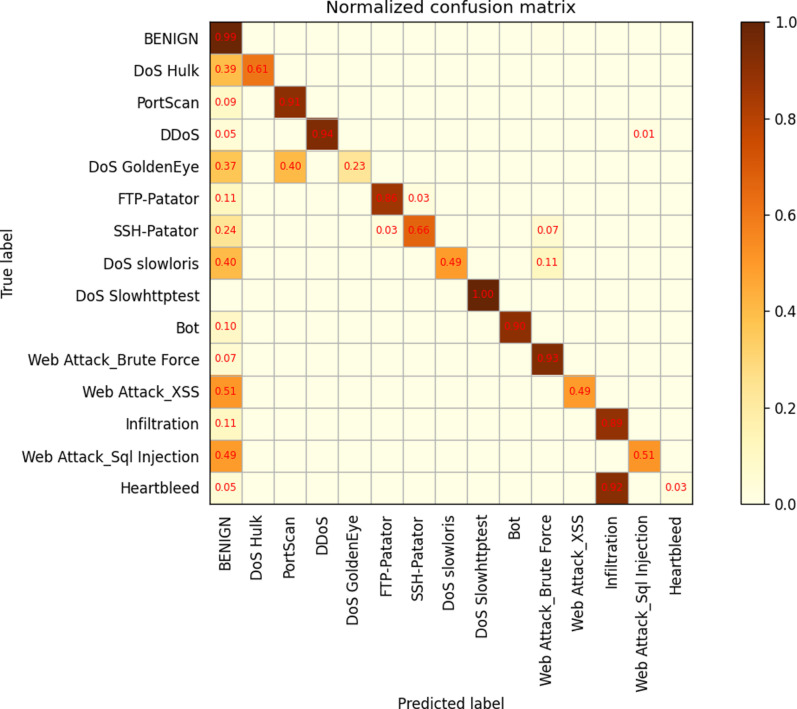
ResNetCBAM101.

In order to further analyze the performance of different layers, a confusion matrix was generated for the 34-layer model. The results show that there are numerous misclassifications across different labeling categories, indicating that the model may not have been trained with sufficient depth. Subsequently, themodel was retrained with 50 and 101 layers. The confusion matrix reveals that while the misclassification rate decreased significantly for the 50-layer model, it slightly increased for the 101-layer model when compared to the 50-layer model,as depicted in Figs 9–11.

**Fig 12:**
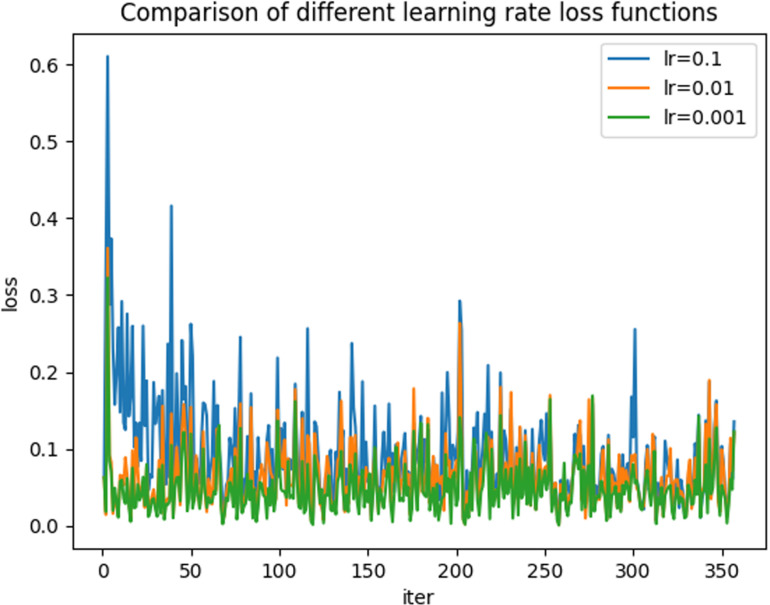
Comparison of different learning rate loss functions.

Based on the comparison results, it is evident that the 50-layer model demonstrates the best overall performance. Subsequently, the parameters of this model are adjusted to further optimize its effectiveness. First, the learning rates of the models are compared, as illustrated in Fig 12. The figure indicates that a learning rate of 0.001 yields the most effective convergence.

**Fig 13:**
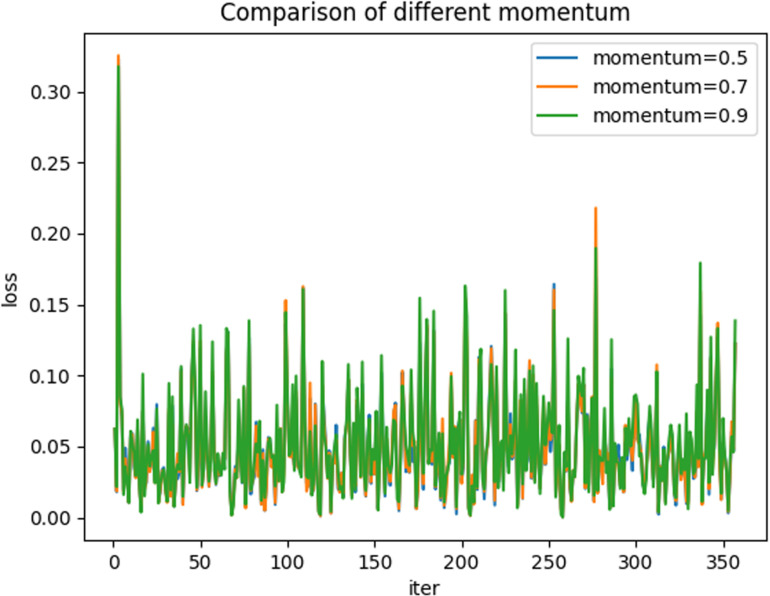
Comparison of different momentum.

Based on the results, we adjusted the parameters of the vector. As illustrated in Fig 13, the convergence of the model under various vector parameters is approximately consistent. This indicates that the opening parameters have a minimal impact on the model’s performance.

The study compared the model’s accuracy, precision, recall, and F1 value for different layers based on the confusion matrix to determine the most suitable number of layers. The confusion matrix provided insights into true positives (TP), false positives (FP), true negatives (TN), and false negatives (FN). Performance metrics such as accuracy were calculated to evaluate the model’s predictive capability.


$$Accuracy=\frac{TP+TN}{TP+TN+FP+FN}\;\;\times \;\;100\%$$ (8)

Precision: This metric indicates the confidence level of a search


$$Precision=\frac{TP}{TP+FP}$$ (9)

Recall: this indicator represents the completeness of the search


$$Recall=\frac{TP}{TP+FN}$$ (10)

F1_score is the weighted average of precision and recall, and this metric indicates the comprehensive performance of retrieval.


$$F1\_score=\frac{2\cdot Precision\cdot Recall}{Precision+Recall}$$ (11)

The comparison results are shown in Table 2 below.

**Table 2:** Comparison of models with different layers.

	Accuracy	precision	recall	F1_score
ResNetCBAM34	99.69%	37.87%	1.63%	3.13%
**ResNetCBAM50**	**99.83%**	72.26%	**66.86%**	**69.45%**
ResNetCBAM101	99.82%	**75.57%**	63.36%	68.93%

The results in the table demonstrate that layer 34 has a recall of 1.63% and an F1 value of 3.13%. On the other hand, layer 50 shows significant improvement when compared to layer 101, with a recall of 66.86% and an F1 value of 69.45%, representing an increase of 3.5% and 0.52% respectively. This improvement enables the model to effectively identify abnormal data in data traffic, predict upcoming attack traffic, and classify the type of attack for appropriate defense. The attention mechanism enhances the model’s ability to prioritize abnormal traffic and reduce detection errors. The experimental results indicate that resnetCBAM50 outperforms traditional models in terms of accuracy. The introduction of the residual module addresses the issues of gradient disappearance and gradient explosion that can arise as the number of layers in the model increases. Consequently, whether the model consists of 50 layers or 101 layers, it can maintain high-level representations while simultaneously improving accuracy to some extent.The model metrics can be found in the table below Table 3. Although the time complexity of the traditional model has increased to some extent, significant improvements have been observed in precision, recall rate, F1 value, and other performance metrics.

**Table 3:** Comparison of model training effects.

	Accuracy	precision	recall	F1_score	Time(s)
CNN	99.80%	**92.15%**	37.62%	53.43%	552.923
LSTM	88.04%	23.80%	20.12%	21.80%	493.233
CNN-LSTM	99.81%	67.90%	62.54%	65.11%	610.498
GRU	86.52%	18.70%	3.64%	6.09%	505.783
**ResNetCBAM50**	**99.83%**	72.26%	**66.86%**	**69.45%**	**970.605**

### 4.4 Experiments based on Maple-IDS

**Fig 14:**
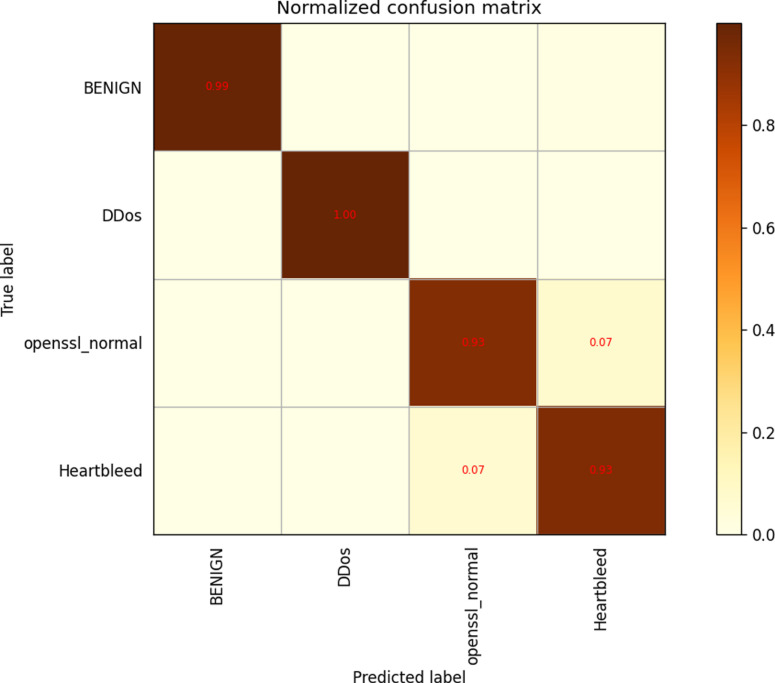
Maple-IDS dataset confusion matrix.

In this study, we incorporated our self-constructed dataset, Maple-IDS, into the ResNet-based model. Through experimentation, we compared the performance metrics of training models using Maple-IDS and CIC-IDS-2017. As shown in Fig 14 Our findings indicate that the prediction accuracy of the self-built dataset is superior when using the same experimental model. This can be attributed to the lower data complexity of Maple-IDS, which ultimately reduces the prediction difficulty for the model.

## 5 Conclusion

With the advent of the Internet of Everything era, cybersecurity has emerged as a crucial area of focus. There is a growing global demand for network situational awareness, highlighting its significance in safeguarding information security and cybersecurity [24,25]. The widespread use of the Internet has further complicated cybersecurity challenges. Consequently, the development of a predictive model capable of swiftly identifying and accurately forecasting cyber threats has become a shared objective in both industry and academia. Such a model holds vital strategic importance in the prevention and mitigation of cyber threats. This paper introduces a resnet-based combined attention mechanism model designed to forecast potential attack methods based on recent data streams. The process involves data preprocessing, model input, and comparison of experimental outcomes. Initial comparisons of the model’s performance across different layers revealed that the 50-layer model exhibited the highest accuracy and rapid convergence. Subsequent validation of the model’s efficacy involved comparing its accuracy with that of traditional models. However, an analysis of the confusion matrix indicated areas for improvement in the model’s predictive capabilities and overall accuracy.

The follow-up work primarily focuses on optimizing the model and processing datasets, with the aim of enhancing predictive model performance. Through the research process, it was observed that data preprocessing and feature extraction play crucial roles in cybersecurity situational analysis. The accuracy of predictions is largely influenced by the quality of data processing and feature extraction [26]. As the volume of data increases, a substantial amount of complex data flows into the network, significantly affecting the real-time performance of security predictions. To mitigate this impact, we propose utilizing big data analysis technology to enhance the processing of data sets, thereby minimizing its effect on real-time performance.Moving forward,we are considering the implementation of Neural Architecture Search (NAS) to dynamically adjust the model’s parameters in order to adapt to rapidly changing network environments. Furthermore, we plan to introduce adversarial samples during training to enhance the model’s robustness, thereby improving the accuracy of model detection.
